# The Challenges of 21st Century Neurotoxicology: The Case of Neurotoxicology Applied to Nanomaterials

**DOI:** 10.3389/ftox.2021.629256

**Published:** 2021-02-18

**Authors:** Anna Bencsik, Philippe Lestaevel

**Affiliations:** ^1^Anses Laboratoire de Lyon, French Agency for Food, Environmental and Occupational Health & Safety (ANSES), Université de Lyon, Lyon, France; ^2^Pôle Santé-Environnement, Service d'Etudes et d'expertise en Radioprotection, Institut de Radioprotection et de Sûreté Nucléaire, Fontenay-aux-Roses, France

**Keywords:** engineered nanomaterials, neurotoxicology, characterization, risk assessment, AOP, human health, protein corona, substance prioritization

## Abstract

After a short background discussing engineered nanomaterials (ENMs) and their physicochemical properties and applications, the present perspective paper highlights the main specific points that need to be considered when examining the question of neurotoxicity of nanomaterials. It underlines the necessity to integrate parameters, specific tools, and tests from multiple sources that make neurotoxicology when applied to nanomaterials particularly complex. Bringing together the knowledge of multiple disciplines e.g., nanotoxicology to neurotoxicology, is necessary to build integrated neurotoxicology for the third decade of the 21st Century. This article focuses on the greatest challenges and opportunities offered by this specific field. It highlights the scientific, methodological, political, regulatory, and educational issues. Scientific and methodological challenges include the determination of ENMs physicochemical parameters, the lack of information about protein corona modes of action, target organs, and cells and dose– response functions of ENMs. The need of standardization of data collection and harmonization of dedicated neurotoxicological protocols are also addressed. This article highlights how to address those challenges through innovative methods and tools, and our work also ventures to sketch the first list of substances that should be urgently prioritized for human modern neurotoxicology. Finally, political support with dedicated funding at the national and international levels must also be used to engage the communities concerned to set up dedicated educational program on this novel field.

## Introduction

Among chemical substances, those which are characterized by a size of <100 nm (at least for one of their three dimensions), belong to the class called nanomaterials. Due to their size these substances have many unique and specific physicochemical properties whether they are optical, magnetic, biological, etc., that differ from those the same chemical substance exhibits in a bulk state. These properties are well-controlled in the case of engineered nanomaterials (ENMs) and can be even further functionalized very precisely. This explains why during the last two decades, ENMs have been produced on a very large scale for various types of applications in consumer as well as industrial products, including energy, electronics, textile, food and agriculture, environmental science, cosmetics (Hussain, 2018; Kaphle et al., 2018; Fytianos et al., 2020). In the biomedical applications, ENMs yield a significant improvement notably in brain disease diagnosis (bioimaging) and treatments, e.g., in cancer therapy or use of nanomaterials as drug or gene delivery vectors (Huang et al., 2017). Due to their small size they permit easy crossing of the biological barriers and, most remarkably, even those protecting the brain (Furtado et al., 2018). However, crossing the blood-brain barrier (BBB) is not the only promising alternative to deliver substances into the brain. Currently, intra-parenchymal or intracerebroventricular injections, non-invasive intranasally administered, are among the strategies used to bypass the BBB. Intranasal delivery takes advantage of the natural pathways used by nanosized particles to reach the brain, including the olfactory sensory neurons and the trigeminal nerve pathways as well as extracellular diffusion (Bencsik et al., 2018).

Unfortunately, several *in vivo* and *in vitro* studies are now also providing evidence of neurotoxic effects of many types of nanomaterials, made of organic or inorganic nanoparticles, and therefore the advantages of ENMs such as those developed for the nanomedicine field but also used in other consumers products, must be weighed against their potential negative effects. The human exposure to ENMs is continuously growing with the rising industrial production of various daily products. From these marketed products, ENMs can enter the human body and may reach the nervous system by many paths—through the systemic compartment after a nano-based drug injection and through the inhalation (respiratory and olfactory) route, especially in the context of occupational exposure, or through the skin, e.g., by the dermal application of cosmetics and by ingestion of food containing nanoadditives. Specific knowledge and mechanisms underlying the uptake, translocation, and fate of ENMs in the nervous system can be found in recent reviews where they are discussed in detail (Bencsik et al., 2018; Boyes and van Thriel, 2020). Remarkably, there is a lack of evidence regarding ENMs' possible adverse health effects on the brain. With respect to the various types of nanomaterials, there is still a shortage of data and regulatory tests to feed risk assessment activities. In addition to considering anthropomorphic sources, if we add the high variety of sources of nanomaterials that can also be natural and thus not only intentionally produced by man, this particular class of chemicals clearly becomes one of the most challenging in the field of neurotoxicology.

This article focuses on the greatest challenges and opportunities offered by this specific field and highlights the specificities related to the nanotoxicology as well as the scientific, methodological, political, regulatory, and educational issues.

## Challenges Related to the Emergent Nature of Nanomaterials as Potential Neurotoxicants

**Figure 1:**
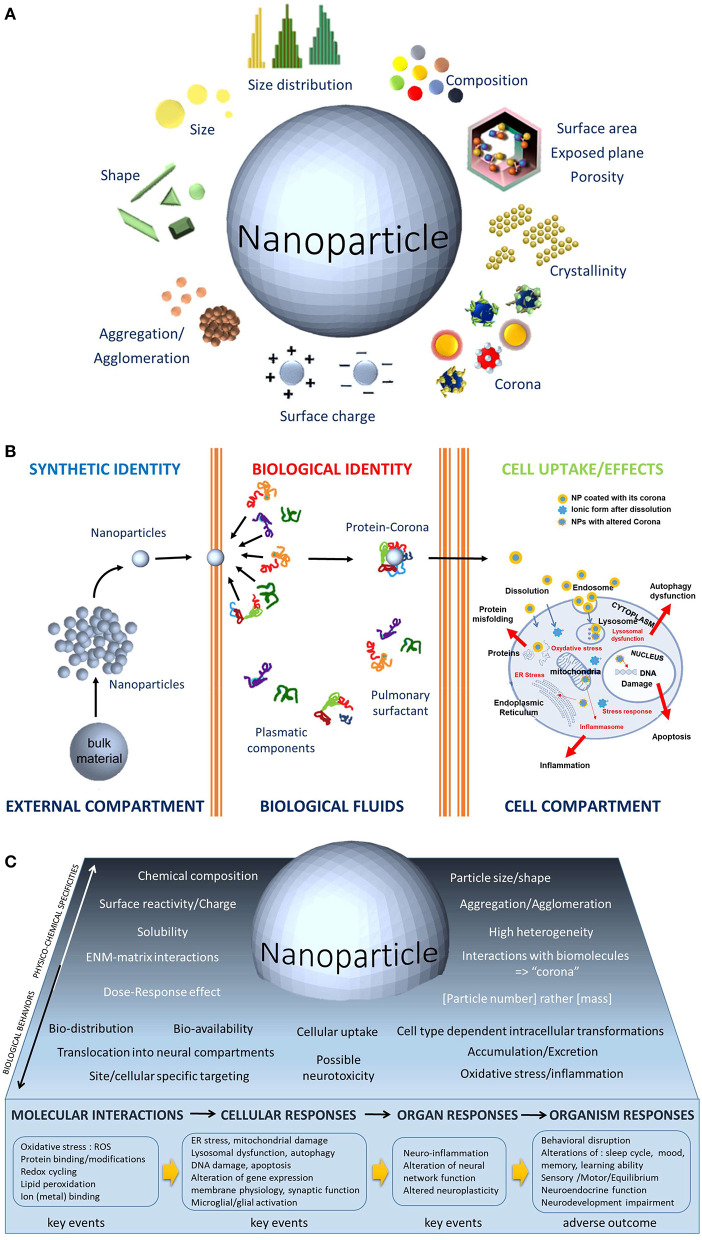
**(A)** The physicochemical parameters of ENMs, such as size, shape, size distribution, composition, aggregation, surface charge, corona, crystalline nature, and porosity, are unique features. The biological activities of ENMs depend on the physicochemical parameters usually not considered in toxicological studies. Because of this dependence, the toxicological studies of ENMs requires a dual approach: (1) a physicochemical one that allows the measurement of the impact of biological environments on the ENMs, notably to evaluate the dissolution ability of the ENMs that would enable further interactions with the cells and (2) a toxicological one that assesses the impact of ENMs on biomolecules, on cells, organs, organisms, and ecosystems. **(B)**. Contrary to the bulk chemical substance that may enter into contact with biological fluids or not, the NPs have a greater chance to enter the body thanks to their nanosize. Once in the biological compartment, a protein corona instantaneously recovers the NPs. Made of biomolecules, their composition depends on the site of entry and the organ considered, (e.g., surfactant proteins in the lung or plasmatic proteins in the blood vessels), this corona gives a new biological identity to the NPs. The corona composition may influence surface charge, dispersity, solubility, nanoparticle transport and thus biodistribution, cellular binding and uptake, internalization process, bioclearance. Inside the cell, the biological entity represents the key event in the adverse outcome pathways (AOP) concept. It triggers successive interactions at the molecular and organelle levels and leads to cellular responses, most often related to oxidative responses and inflammation. **(C)**. Graphical abstract that illustrates the distinct complexity of neurotoxicology applied to nanomaterials. The lower part exemplifies the KEs and the subsequent AOPs related to biomodification resulting from nervous system exposure to NPs.

The special class of chemical substances characterized by their nanodimension, massively produced and put on the market for several decades now needs particular attention with regards to their neurotoxic potential. Remarkably, compared with their bulk counterparts, these chemical substances at the nanoscale possess novel properties that can be optical, magnetic, electronic, and mechanical and have higher chemical reactivity as well (Auffan et al., 2009). Looking at these features from a toxicological point of view, key questions arise, as the nanoscale is within the range of the size of cell membranes and intracytoplasmic components. Consequently, the basis of the understanding the cytotoxicity of nanoparticles (NPs) relies on the ways by which NPs are taken up, what their sub-cellular distribution is, how NPs interact with the different intracellular components, with the organelles and ultimately with the DNA and how there are metabolized and/or excreted. Another major question is, for a given chemical substance, whether the toxic capabilities are different at the nanoscale. What kind of physicochemical features that define NPs as illustrated in Figure 1A would then govern toxicity? Is there a size-, shape-, charge-, or surface reactivity; crystalline changes; or any other parameter dependence that would sustain the mechanisms of toxicity of these nanoscale chemicals?

All these questions are of utmost importance to assure production of new safe nanomaterials that could be then designed to keep the novel properties intact, which will allow advances in so many different fields of application. These questions led to the nanotoxicology field as well as the emerging concept of safety by design that considers the whole life cycle assessment to optimize the benefit/risk ratio. It integrates the societal and economic risks as well, which is relatively new compared with previous technologies.

In theory, neurotoxicological studies of the health effects of nanomaterials should not be inherently different from that of other chemicals, but many parameters associated to nanoscale (Figure 1A) make such studies new and exigent—for instance, the nanomaterials are not simply chemical molecules but are also physical objects with specific properties, most notably having a large hyper-reactive surface.

Indeed, it seems that the physicochemical behaviors, thermodynamics, toxicology, and ecotoxicology of NPs are all properties that cannot be extrapolated directly from those of larger solids or molecular compounds. Beside the particle size, the surface reactivity is probably one of the most important parameters to consider of these toxicological abilities, since surface reactivity is negligible in microsized particles and not in NPs (Auffan et al., 2010). The critical diameter size below which specific nano-effects occurs appears most often to be under 30 nm. It is correlated with a greater number of atoms localized at the surface of the chemical substance (Auffan et al., 2010). The reactivity and biological effects of NPs of a given chemical substance can be size-dependent and shape-dependent (Morones et al., 2005; Pal et al., 2007), but surface charge and shape are also able to trigger specific interactions and have a biological effect (Kim et al., 2016). In addition, compared with fine particles made of the same material, NPs appear to be less efficiently cleared (Oberdorster et al., 1994). Therefore, it is obvious that when examining the question of neurotoxicity of nanomaterials, it is necessary to consider these specific points that are compulsory in classical nanotoxicology studies.

There are several other particularities to know when considering ENMs toxicity and as a result, the challenges are high, notably in how to establish the identification, characterization, and even the classification of their neurotoxic abilities. The high heterogeneity of ENMs, in terms of the many physicochemical parameters as well as their production conditions, their presence in different matrices in the final marketed products (e.g., for TiO_2_ NPs, within food, cosmetics, and paints) adds complexity. It appears very difficult for a category of nanomaterials to be predictive; instead, variety is the main rule for nanomaterials. Even if NPs share the same chemical composition, they can produce distinguished biological responses and thus must not be taken as a unique type of nanomaterial as illustrated for TiO_2_ NPs (Kose et al., 2020). Since there are hundreds of different nano-TiO_2_ compositional and functional differences (Luttrell et al., 2014; Harris et al., 2020), allowing different applications, e.g., as UV filters in cosmetics such as in sunscreen creams, whiteners in food with the E171 food additive, etc., it is not possible to conclude or make a stereotyping toxicology for a form of NP. On the contrary, each type of TiO_2_ NPs may produce differential hazard effects in neurotoxicity studies, and it is better to stick to specific examples and to avoid generalizing. Thus, a rigorous and complete characterization of the test substance (Figure 1A) is an important prerequisite in any neurotoxicological study.

It is even more important that there is a lack of standardization in the methodological approaches especially to assess the nano-neurotoxicity (Bencsik et al., 2018). Combined with the lack of toxicologically well-characterized particles as positive and negative “Benchmark Materials,” it generates data that are apparently contradictory, which are in fact simply incomparable. Another issue to consider is a matter of debate; about the most suitable dose metric to use in the nanotoxicological studies, notably whether to use the mass, particle number, or surface area concentration to assess the response of cells or organisms to NP exposure. At the nanoscale, the surface area or the particle number concentration could be a most relevant descriptor of toxicity (Petersen et al., 2019). The expressed mass concentration would fail to correctly predict the biological effect of NPs. In a general manner, there is a lack of information and comprehension of ENMs modes of action and likely dose–response function. It appears that the size, the shape, and the surface chemistry interact with the dose to define non-linear response patterns (Bell et al., 2014). Cautious dose evaluations are thus necessary for significant risk assessments of ENMs and to allow a major contribution in regulatory procedures.

Another level of complexity to consider when dealing with the neurotoxicology of nanomaterials is to pay attention to the result of the first interactions of the nanomaterials with biomolecules. Once the nano-objects come into contact with biological media (extra and intracellular fluids) they do not remain naked but are instantaneously recovered with one or more layers of biomolecules (Figure 1B) —mainly proteins—to form at their surface a dynamic cloud of proteins called “corona” (Walczyk et al., 2010). The composition of this corona varies with time, and the locations of the nanomaterials do so too, notably during traveling from one organ to another, and through extra- and intracellular traveling (Pisani et al., 2017). Thus, the corona is a dynamic and time-dependent process, and its composition will be different in the central nervous system compared, e.g., with the blood compartment (Shim et al., 2014). It is critical to understand that what neural cells are first “seeing” is not the nanomaterial itself but a new “biological identity” (Figure 1B) made of the corona around the NPs (Walczyk et al., 2010). The biological behavior of the ENMs, their biodistribution, cellular uptake, intracellular transformation, clearance, excretion as well as their possible toxicity will be determined by this corona (Figure 1B). Elucidating corona formation is thus of upmost importance and is one of the most challenging questions in nanotoxicology—and in the neurotoxicology of nanomaterials as well. First, the biological identity will govern the way to pass through the cell membrane, by dissolution, phagocytosis, macropinocytosis, receptor-mediated uptake, internalization mediated either by clathrine-dependent or caveolae-mediated endocytosis depending on particle size (Rejman et al., 2004). Once inside the cell, the biological entity represents the first key event (KE) in the adverse outcome pathways (AOP) concept as summarized in Figure 1C. It triggers successive interactions at the molecular (ROS production, lipid peroxidation…) and organelle levels (lysosomal dysfunction, endoplasmic reticulum stress, etc.) and may lead to cellular responses, most often related to oxidative responses and inflammation, (e.g., microglial activation and alteration of synaptic function in neurons) and then to organ responses such as neuroinflammation, neurotransmission/neuroplasticity alterations. Finally, these KEs may lead to adverse outcomes at the organism level expressed, e.g., by behavioral disruptions, neurodevelopment impairments, etc.

There is a lack of information about ENMs modes of action on the nervous system and it is very difficult to define a potential dose–response function. It is our opinion that deleterious effects might happen, even more probably if the exposure is repeated and chronic, even if the doses considered are low. Unfortunately, most neurotoxicological studies used short exposure durations, often coupled to high exposure levels, while humans are essentially chronically exposed to low levels. There are thousands of potential neurotoxicants that remain untested in humans (Grandjean et al., 2010; Grandjean and Landrigan, 2014). The cumulative effect of multiple exposures may be above the safe regulatory dose, while each exposure is low (Schecter et al., 2013). Therefore, there are two main challenges to overcome in assessing the neurotoxic hazard for ENMs: (1) to define a prioritization among ENMs and (2) to find alternatives to *in vivo* experiments. A first selection can be based on exposure levels that would require better monitoring that will probably be available in the next decade (Peters et al., 2020; Salou et al., 2020). This is a crucial point, as most experimental exposures used so far are rarely realistic for human exposure. Prioritization must rely on the aim to protect the most susceptible populations and exposures to ENMs occur during gestational/neonatal and childhood periods, but also on aged populations that are more prone to develop neurodegenerative diseases. Some priority nano-substances for 21st century neurotoxicology are summed up in Box 1.

Box 1 **Priority nano-substances for the 21st century neurotoxicology.**

- Air pollution is a major exposure pathway of neurotoxicants, and among the particulate matter, NPs are the largest contributor to the air pollution (Rönkkö and Timonen, 2019).
- Nano-pesticide intoxication is one of the most serious threats to human health. Given the extensive use and exposure to (nano)-pesticides, the general lack of data on neurotoxicity is a serious problem (Iavicoli et al., 2017).
- Neurotoxicity remains a subject to resolve within many nano-systems such as nano-emulsion or nano-capsule used in cosmetic products (Prashant, 2017).
- The next “hot subject” in neurotoxicity might also come with the presence of nano-plastics in food, water, and even in the air. Despite the ubiquitous presence of nano-plastics in the environment, there are very few data regarding their neurotoxicity (Prüst et al., 2020).
- Many metal NPs have numerous applications that increase the risk of their neurotoxicant effects (Bencsik et al., 2018; Teleanu et al., 2019; Boyes and van Thriel, 2020). Although there are several available neurotoxicity assessments of these metal NPs, there is a deficiency of standardized and consistent neurotoxicological studies.

The future of neurotoxicology applied to nanomaterials must include the development of different predictive models at different scale. At first for NPs' corona formation and composition, it is important to understand its evolution with time as well as its influence on the attachment and uptake by neural cells, as well as on the biodistribution, bioprocessing, and bioclearance, especially within the brain. It would be necessary to revise the validated neurotoxicological tests of the Organization for Economic Cooperation and Development (OECD), e.g., by introducing more complex models of cocultures based on various cell types of the nervous system and those derived from human pluripotent stem cells, which remove the difficulty of extrapolations between species (Pamies et al., 2017; Dreser et al., 2020). As well as having a high degree of correlation with mammalian nervous systems, the nematode *C. elegans*, the *drosophila* and the zebrafish are useful alternative neurotoxicological models for behavioral tests (Peterson et al., 2008).

It is our opinion that future directions for neurotoxicology research applied to nanomaterials also include the elucidation of initiating KE and the subsequent AOPs related to biomodification resulting from exposure to NPs. AOPs have emerged as a new framework to predict toxic outcome using molecular level effects (Edwards et al., 2016). Because they are not chemical-specific and they improved predictions of neurotoxicity *via* decreased uncertainty and increased transparency (Bal-Price and Meek, 2017), they are critical for risk assessments. The AOPs informed/enhanced species-to-species extrapolation and can be life-stage specific. To improve AOPs' usability even more, the next step is to develop quantitative AOPs and to link AOPs to regulatory endpoints and test guidelines and to develop guidance/framework for reporting and applying AOPs for regulatory applications. The website dedicated to AOPs (AOP Wiki: https://aopwiki.org/) created by the OECD provides a knowledge-based tool, constantly in development and refined, bringing together all knowledge on how chemicals can induce adverse effects. If some domains start to be well-documented, neurotoxicity is associated to three AOPs only and no KEs despite the many data published in neurotoxicology. It shows how important it is to sensitize the entire neuroscientific community to the necessity to learn the existence of this tool and propose some AOPs and KE respective the process provided by OECD (ilibrary, 2018).

The next perspective for this specific field of toxicology can be found in the new predictive approaches such as those offered by the toxicogenomics, bioinformatics, systems biology, and computational toxicology, introducing the use of machine learning tools to build predictive models for the toxicity of ENMs (Mahadevan et al., 2011; Smirnova et al., 2018; Bahl et al., 2019; Nussinov et al., 2019; Thomas et al., 2019). Applicable also to developmental toxicity (To et al., 2019), they all provide outstanding opportunities to overcome in an unprecedented manner the complexity of combining neuro- and nanotoxicology. In addition, it will feed exposome science (Wild, 2005), which requires the combination of high dimensional biology and system science aiming at integration using big data analytics and bioinformatics (Tamayo-Uria et al., 2019). The exposome has yet to be applied to the etiology of brain health disorders.

As neurotoxicological data obtained in the past for larger particles of “classical chemical” may no longer be valid for the nano-forms, these series of novel strategies are being advanced to identify properties that best predict ENMs' risk potential. To be valid at a regulatory level, it is necessary (1) to collect the information on the physico-chemical properties, biopersistence, and reactivities (2) to provide compliance with Good Laboratory Practices (“GLP”), testing results such as acute and chronic toxicity, dosing information such as bio-kinetics, exposure levels, frequencies, and duration of exposure to consumers, workers, and the general population. The success in all these challenges will also rely on an educational strategy oriented toward neurotoxicology of the nanomaterials that bring along with their special challenges. In preparation for the expected workload increase for neurotoxicity testing on chemicals and physicals, it will be necessary to sufficiently expand the number of neurotoxicologist and econeurotoxicologist experts, too.

## Conclusion

For the 3rd decade of the 21st century, progress in the neurotoxicology of nanomaterials appears as one of the most challenging processes, and progress is expected on scientific issues that require fundamental and applied research, e.g., to understand the formation of the corona, as well as the fate of ENMs within the neuronal tissue, and to characterize and classify the different ENMs according to their neurotoxicity. Progress is also constantly needed with respect to the methods used, such as metrological and biomonitoring tools: measuring individual exposure, identifying, visualizing, and quantifying ENMs within human nervous system; new integrative models (IATA); validation of non-mammalian models for neurotoxicological studies, computational tools, epidemiological tools investigating their specific long-term brain health effects for occupational workers, the general population, the elderly vs. the young, and developing countries vs. advanced countries as well. We think that the success of these challenges entails cooperative work and the implication that can be financial or intellectual, between authorities, industries, and interdisciplinary scientists from various fields including chemistry, physics, chemical engineering, neurosciences, and computational sciences, etc. Among the challenges of 21st century neurotoxicology, it is time to improve the neurotoxicology of nanomaterials.

## Data Availability Statement

The original contributions presented in the study are included in the article/supplementary material, further inquiries can be directed to the corresponding author/s.

## Author Contributions

AB and PL wrote the paper. AB conceived the figures. All authors contributed to the article and approved the submitted version.

## Conflict of Interest

The authors declare that the research was conducted in the absence of any commercial or financial relationships that could be construed as a potential conflict of interest.
